# Syphilitic Cases from Private Practice

**Published:** 1882-08

**Authors:** P. O’Connell

**Affiliations:** Sioux City, Iowa


### Notes from Private Practice.

ARTICLE III.

SYPHILITIC CASES FROM PRIVATE PRACTICE. By P. O'CONNELL, M.D.

The following short notes may help to show how erratic and uncertain, as regards time, may be the manifestations of syphilis:

On December 14, 1874, Lilly Mary T., aged fourteen days, at whose birth I attended, and who was free of disease until now, came under my care for impetigo syphilitica. Her mother had had treatment during utero-gestation, though ignorant of her disease, owing to my having seen some condylomata about her mouth one year ago. The father, too, was regularly under treatment and observation. Inunction alone was carried out on the child, and by January 6, 1875, was again well. When just a year old I vaccinated her, and the vaccination was perfectly normal. In February, 1877, she had scarlatina simplex. From December, 1874, to July 4, 1878, when I prescribed for syphilitic headache, there was no evidence of the disease.

On February 11, 1877, and again on October 3, 1879, I delivered the mother, on each occasion, of a living daughter, free of all evidence of the disease. From September 14, 1875, to February 25, 1881, there were no manifestations of syphilis in the mother. Meantime the father continued to manifest the disease from time to time, although under treatment.

Four years after birth the second child showed the first lesion—a roseola syphilitica. At the same time the father had a "skin rash." My information is now obtained from a letter written by the father to me.

The father got his pox about New Year's, 1872. While suffering from the primary lesion, and being under promise of marriage, some wretched man, so-called a physician, advised him to fulfill his promise. This he did, and, within a year, his wife gave birth to an infant, with all the evidence of the disease, and which survived only a few days.

On September 7, 1874, John M., aged twenty-nine years, came under observation. He was extremely emaciated; both legs were quite black; he could neither walk nor stand; hands and feet cold and blue; right eye protruded, staring, motionless—there is paralysis of the third, fourth, sixth, and portio mollis of the seventh nerves; pupils dilated and fixed; speech long, slow, difficult. Was delirious most of the time, especially so at night, when he attempted to walk about. When upright, he walks a few steps, staggers, and falls heavily, where he remains until raised. He falls out of bed on attempting to get out of it. No sensation in the rectum; no control over the sphincters. Is the picture of wretchedness.

Inunction and iodide potassium, with good diet, including whisky, constituted the treatment. By September 30, the eye was retracted and movable; hearing good. He was up and able to move about the house, and could converse freely. Was free of headache, which he says was intense.

The following facts he now communicated to me: He contracted his disease twelve years ago. About July 17 last left hemiplegia came on; he was insensible for three days, and speechless for three weeks. Had some half-dozen fits, the first in May of this year. Had headache for seven years, and always on the right side; it was extremely severe at the time I first saw him, he said. Perhaps this was due to morphia, which he got from a quack, who treated him before me.

His only remaining trouble now was the want of control over the rectum, which was not improved in the least. His appearance was vastly improved. At this stage I lost sight of him. But his mother, whom I met on the street, informed me that he went into the hospital for the bowel trouble; that all his symptoms rapidly recurred and ended in his death in the hospital on

November 25, just two months from the time he stopped my treatment. There was no autopsy.

On August 8, 1875, I saw Mrs. K., aged forty years, and married to her second husband, by whom she was infected, nearly eight years. Since the birth of her last child, four years and three months ago, she has been in bad health. She is aware of the nature of her disease, and says the first lesion noticed by her was sore throat four years ago. Two years and two months ago she got cranial nodes; one and a half year ago some dead bone was removed from the forehead; one year ago some dead bone came away from the left supra-orbital ridge; five months ago resection of the right elbow was performed for caries of the olecranon. There is pain, tenderness and swelling over the nasal bones, and three nodes (soft) on the skull. Nocturnal headache preventing sleep. The thyroid is uniformly enlarged to the size of an ordinary orange, very hard, closely grasping the trachea, rendering respiration very difficult—markedly dyspnœal and stridulous. The trachea is quite tender under the gland. The enlargement began soon after the sore throat was noticed, and has steadily increased since.

Tincture and liniment of iodine, equal parts, externally, and liberal doses of iodide potassium internally, quickly reduced the gland, and I lost sight of her. But I learned on reliable authority that she died of intra-cranial syphilitic disease in 1879.

On February 11, 1876, Thomas G., aged forty-seven, in apparent excellent health, consulted me for a "sore on his arm." He has a papular syphiloderm across the forehead close to the hair, with a few papules near the eyebrows, and three most characteristic rupial sores on the outer side of the right forearm just below the elbow. He has not the most remote suspicion that the present "sore on his arm" and frontal rash are syphilitic.

On closely questioning him, he said he had venereal sore (character unknown) first and last in 1850, or twenty-five years ago. It was treated by caustic of some kind and quickly healed. He positively and emphatically asserts that he had no lesion since of any kind. Green iodide mercury, gr. 1 in pill, every night for a short time, followed by corrosive sublimate and iodide potassium completed the cure by March 13.

Annie G., daughter and only child of the above, aged eight years, a strong, hardy, active, and hitherto healthy child, suddenly sickened at 5 p. m. on November 23, 1873, with severely acute occipital headache, which soon became general. Active delirium, with very severe and prolonged convulsions set in, followed by effusion and death in thirty hours. No autopsy. The sudden onset of the fatal meningitis, for which no cause could be found, puzzled me much. Now I assume, rightly or wrongly, that syphilis caused it.

Mrs. W., aged thirty-five years, infected by her husband eight months ago, came under my care on April 26, 1876. During the six weeks following the date of infection, she said she had chills and fever by day, and especially at night, and a "very disagreeable, nasty looking rash," which, from her description, would seem to be ecthyma. Then followed chancre on the genitals, when the chills and fever ceased. Sore throat followed. A great deal of the hair of the head, and some of her finger-nails fell off. Then came vulvar condylomata, which healed one month ago. Had had treatment. On the right olecranon is a superficial rupial scab, from under which flows a stinking sanies. Her chief complaint is of a terrible headache for a month, with "giddiness and lightness," and a fear of becoming insane. Knows the nature of her disease. Prescribed green iodide mercury, corrosive sublimate and iodide potass. during May and June. She proved to be pregnant at this time, and tock, during her pregnancy, on and off, a mixture of iodide potassium, corrosive sublimate and tinct. cinchonæ flav., and was delivered, on January 18, 1877, of a boy nine or ten pounds' weight, and free of any sign of syphilis. On July 30, 1878, I prescribed iodide potass. for the child, who was cross, restless, cried at night, and did not sleep until early morning. After this he was quiet, rested and slept well all night. He improved in health and appearance, and was, seemingly, well by August 29. His canine teeth were characteristic of hereditary syphilis.

Early in June, 1876, I attended Mrs. C., aged thirty years, for a miscarriage, at seven and a half months' gestation. The fœtus had been dead about two weeks before expulsion, and I did not see anything special on its skin nor about the mother to attract notice. On December 14, following, she manifested undoubted syphilitic skin-rash. In March, 1880, she had a living child, at term, without manifest disease. I prescribed hydrarg. bi-chloride during pregnancy. On August 14, I saw her husband with a syphiloderm. He drank a great deal, and had "delirium tremens." He was very careless in carrying out treatment, now and then pushing drugs in a reckless and dangerous fashion. I found it impossible to keep him under steady observation, months sometimes going by without seeing him. Once, while suffering from pain in the head, and in a large tibial node, at night, he took forty-five grains, thrice daily, of iodide potass. for some months. Seeing he could not continue it, and growing worse, he called on me, when I found severe iodism, very naturally. A subperiosteal abscess formed on the tibial node. This I opened, after which it quickly healed. Finally, I lost sight of the man. He eventually died on December 2, 1881, from some intra-cranial mischief, no doubt syphilitic.

Some high authorities, Fournier, for example, tell us that corrosive sublimate, the form which I prefer and usually employ, disagrees, is badly tolerated, or is positively injurious in pregnancy. In my experience (not very small) I find it not only does not increase the anæmia of pregnancy, but, on the contrary, obviates it. Neither could I see that mercury, in any form, internally, caused or increased gastric troubles. I never hesitate to prescribe the metal for syphilitic pregnant women. And I am satisfied that a careful use of it is the best protective against miscarriage such women possess.

SIOUX CITY, IOWA.

